# Home ranges, directionality and the influence of moon phases on the movement ecology of Indian flying fox males in southern India

**DOI:** 10.1242/bio.059513

**Published:** 2023-01-30

**Authors:** Baheerathan Murugavel, Sripathi Kandula, Hema Somanathan, Almut Kelber

**Affiliations:** ^1^ IISER TVM Centre for Research and Education in Ecology and Evolution (ICREEE), School of Biology, Indian Institute of Science Education and Research, Thiruvananthapuram, Maruthamala P. O, Vithura, Kerala 695551, India; ^2^ 74-6-51, Sravanthi Enclave, Prakash Nagar, Rajamahendravaram, Andhra Pradesh, 533103 India; ^3^ Lund Vision Group, Department of Biology, Lund University, Sölvegatan 35, 22362 Lund, Sweden

**Keywords:** Chiroptera, Landscape use, *Pteropus giganteus*, *Pteropus medius*, Pteropodidae, Urban ecology

## Abstract

Flying foxes of the genus *Pteropus* are amongst the largest fruit bats and potential long-range pollinators and seed dispersers in the paleotropics. *Pteropus giganteus* (currently *P. medius*) is the only flying fox that is distributed throughout the Indian mainland, including in urban and rural areas. Using GPS telemetry, we mapped the home ranges and examined flight patterns in *P*. *giganteus* males across moon phases in a semi-urban landscape in southern India. Home range differed between the tracked males (*n*=4), likely due to differences in their experience in the landscape. We found that nightly time spent outside the roost, distance commuted and the number of sites visited by tracked individuals did not differ significantly between moon phases. In 61% of total tracked nights across bats, the first foraging site was within 45˚ of the emergence direction. At the colony-level, scan-based observations showed emergence flights were mostly in the northeast (27%), west (22%) and southwest (19%) directions that could potentially be related to the distribution of foraging resources. The movement ecology of fruit bats in relation to the pollination and seed dispersal services they provide requires to be investigated in future studies.

This article has an associated First Person interview with the first author of the paper.

## Teaser

**Summary:** Using GPS telemetry, we mapped the home ranges and flight patterns of *Pteropus giganteus* males in southern India. We hope these data will also help conservation efforts.

## INTRODUCTION

Home range estimates play a key role in understanding animal movement and spatial ecology (Cranford, 1977; Fedigan et al., 1988; Gil et al., 2014; Smith and Griffiths, 2009). Burt (1943) defines home range as ‘the area usually around a home site, over which an animal normally travels in search of food’. The movement ecology of Old-World fruit bats is important to understand as they are pollinators and seed dispersers in the palaeotropics (Aziz et al., 2021; Fleming, 1982; Fleming et al., 2009; Fujita and Tuttle, 1991; Seltzer et al., 2013). Larger fruit bats including flying foxes in the genera *Pteropus, Acerodon,* and *Desmalopex* (family Pteropodidae), are capable of long-distance movement (Breed et al., 2010; Nowak et al., 1994; Tidemann and Nelson, 2004; Welbergen et al., 2020), and render ecosystem services such as pollination and seed dispersal over large spatial scales (Aziz et al., 2017; Aziz et al., 2021; Nakamoto et al., 2009; Oleksy et al., 2017).

Home ranges have been estimated using radio telemetry in Ryuku's flying fox *Pteropus dasymallus* (Nakamoto et al., 2012), and using global positioning systems (GPS) or global system for mobiles (GSM) based telemetry in Livingstone's fruit bat *P. livingstonii* (Mandl et al., 2022), Lyle's flying fox *P. lyeli* (Choden et al., 2019), the Madagascan flying fox *P. rufus* (Oleksy et al., 2015) and the Mauritian flying fox *P. niger* (Oleksy et al., 2019). Telemetry studies on flying foxes have also yielded information on the seasonal use of tree species for foraging (Abedi-Lartey et al., 2016; Mildenstein et al., 2005; Nakamoto et al., 2009; Weber et al., 2015), on seasonal roosting patterns (Eby, 1991; Palmer and Woinarski, 1999), annual migration (Welbergen et al., 2020) and differences in movement patterns between sexes and age groups (Banack, 1998; Eby, 1991; Field et al., 2016; Nakamoto et al., 2012; Walton and Trowbridge, 1983). Recent studies on movement in Egyptian fruit bats provide insights into the involvement of cognitive map-based navigation (Harten et al., 2020; Toledo et al., 2020), and spatial resource partitioning among colonies (Lourie et al., 2021).

Studies on the movement ecology of flying foxes are largely lacking in the south Asian tropics, including the Indian subcontinent, which has 14 pteropodid species including five species of flying foxes (Bates and Harrison, 1997; Saikia, 2021). Overhead electric lines in urban and rural India and Sri Lanka are a major cause of mortality of flying foxes and such lines are often found along the edges of roads and close to human settlements (Chouhan and Shrivastava, 2019; Tella et al., 2020). Hence, it is important to understand the movement patterns of flying foxes in urban and rural landscapes.

Radio-tracking studies in smaller frugivorous bats have shown that flight patterns differ between moonlit and moonless nights (Morrison, 1978; Nair et al., 1998). In general, they avoid moonlit nights, possibly to minimise risks from visually hunting predators (Morrison, 1978; Elangovan and Marimuthu, 2001). Colony level observations suggest that the timing of flights varies little across moon phases in the large Indian flying fox *Pteropus giganteus* (currently *medius*) (Sudhakaran et al., 2012; Murugavel et al., 2021). One study also reported fewer *P. giganteus* individuals at foraging sites during full moon than new moon nights (Sudhakaran and Doss, 2012). These studies on *P. giganteus* did not examine flight patterns within and between individuals across moon phases. The Indian flying fox *Pteropus giganteus* is a habitat generalist that roosts in undisturbed forests, megacities, and rural agricultural areas (Dookia and Tak, 2004; Mishra et al., 2020). A radio-tracking study on two individuals of this species in Sri Lanka reported a larger foraging range in an adult female compared to an immature male (Walton and Trowbridge, 1983). A more recent GPS tracking study from Myanmar has compared movement patterns of ten *P. giganteus* males for up to 3 months to understand their land use in human-modified landscapes in an epidemiological context (McEvoy et al., 2021).

Here, we have mapped the home ranges and investigated nightly movement patterns across moon phases in *P. giganteus* males using GPS tracking. We hypothesised nightly flight durations, distances travelled and the number of foraging sites visited to be greater on brighter moonlit nights than on darker nights. Since fruit bats are long lived and possess cognitive-map-based navigation (Harten et al., 2020; Pierson and Rainey, 1992; Toledo et al., 2020), we hypothesised that emergence direction of individuals on a given night would be aligned with the direction of the first foraging site. Since foraging resources are likely to be distributed non-uniformly around the roosting site, we also examined whether the colony showed flight directionality following emergence using visual scans to estimate the proportion of emerging bats in eight directions relative to the roost.

## RESULTS

### Nightly movement of individuals

**Fig. 1.**
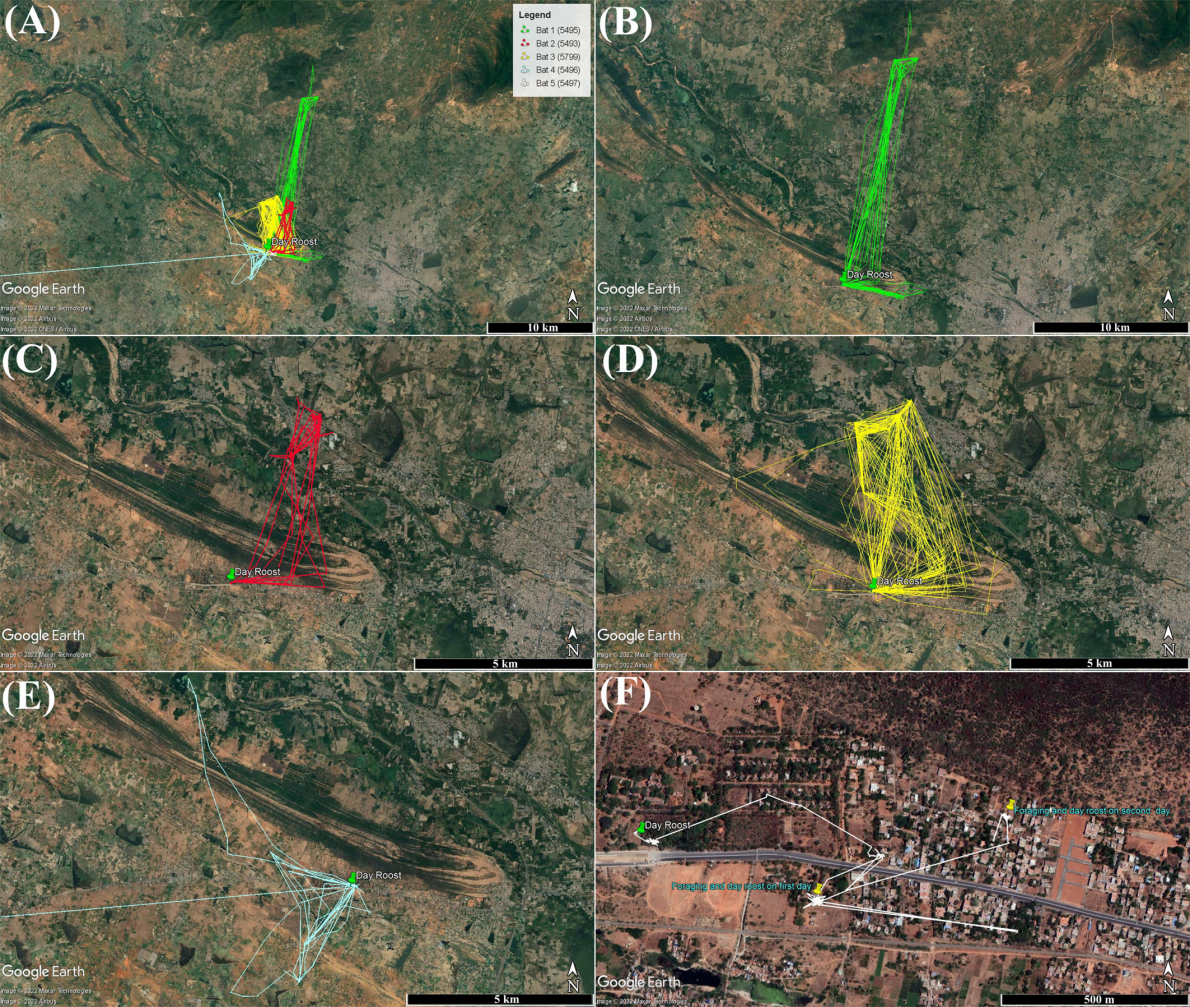
**Foraging tracks of five male Indian flying foxes that provided more than two nights of flight information during the study.** (A) Combined tracks, with each colour representing a single individual. (B) Bat 1 - subadult, (C) Bat 2, (D) Bat 3, (E) Bat 4, (F) Bat 5. Roost location in each map is denoted by a green pointer and other location points represent GPS fixes for the corresponding tracks. The tracks and fixes are overlaid on Google Earth maps.

**Fig. 2.**
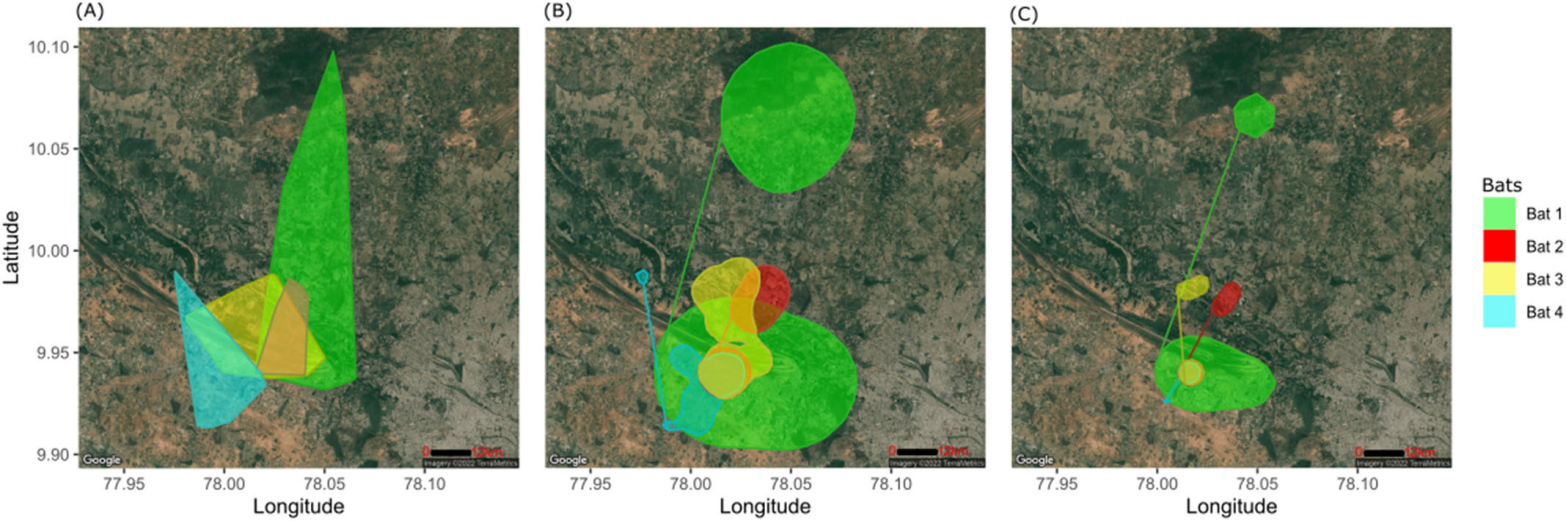
**Home ranges of the four tracked males overlaid on a map of the study landscape.** (A) Minimum convex polygon areas, (B) Core areas based on 95% kernel density estimates and (C) 50% kernel density estimates. Each colour represents one individual. Maps generated from stamen maps using the ggmaps package in R (Kahale and Wickham 2013).

Flight tracks of all five males (*N*=94 nights) that provided data in more than one night are shown in Fig. 1A. Table S1 gives the details of all data retrieved from each individual. Four individuals that completed more than one foraging trip varied in distance travelled, direction, and duration of foraging (Fig. 1A-E and 2A,B). The maximum distance travelled in one night was 42 km, by the sub-adult male (Bat 1) to a reserve forest about 20 km north of the roost (Fig. 1B). The minimum distance covered was 4.3 km, during a foraging trip by the adult Bat 4 (Fig. 1D) to a mango tree in a private garden to the south of the roost that was frequently visited by this individual. Adult Bat 4 was missing from the study roost 48 days after tagging and was found dead 49 days later, in another roost inside a coffee plantation ∼108 km away (∼800 m above sea level; Thankamony, Kerala; Fig. S1).

All four males emerged from the roost within 1 h after sunset, but return times varied between individuals and ranged from midnight to sunrise (Table 1). While adult Bats 3 and 4 spent approximately 5 h outside the roost each night, the sub-adult Bat 1 and adult Bat 2 spent more than 10 h away from the roost (Table 1; Fig. 2A). Bat 2 commuted shorter distances than the sub-adult, like the other two adults, but spent relatively more time at foraging sites (Fig. 2A,B).

**Table 1.**
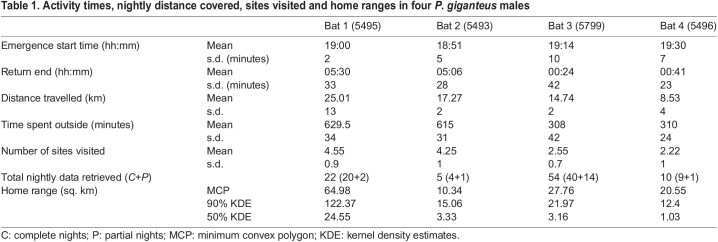
Activity times, nightly distance covered, sites visited and home ranges in four *P. giganteus* males

Bat 5 (the old male) showed an unusual flight pattern (Fig. 1F). It did not make any successful foraging trips during the two nights of data collection and was excluded from these comparisons. It was captured during a return flight, tagged early in the morning around 05:00 h, and was released in the roost by 07:00 h. This animal spent the first day after tagging in the colony, emerged after 22:00 h and did not return to the colony. Two days later, it was identified roosting solitarily in a foraging tree (Fig. 1F). Because of this unusual behaviour, the animal was trapped at its foraging site, the transmitter was retrieved, and it was released into the colony.

### Home ranges and foraging sites

**Fig. 3.**
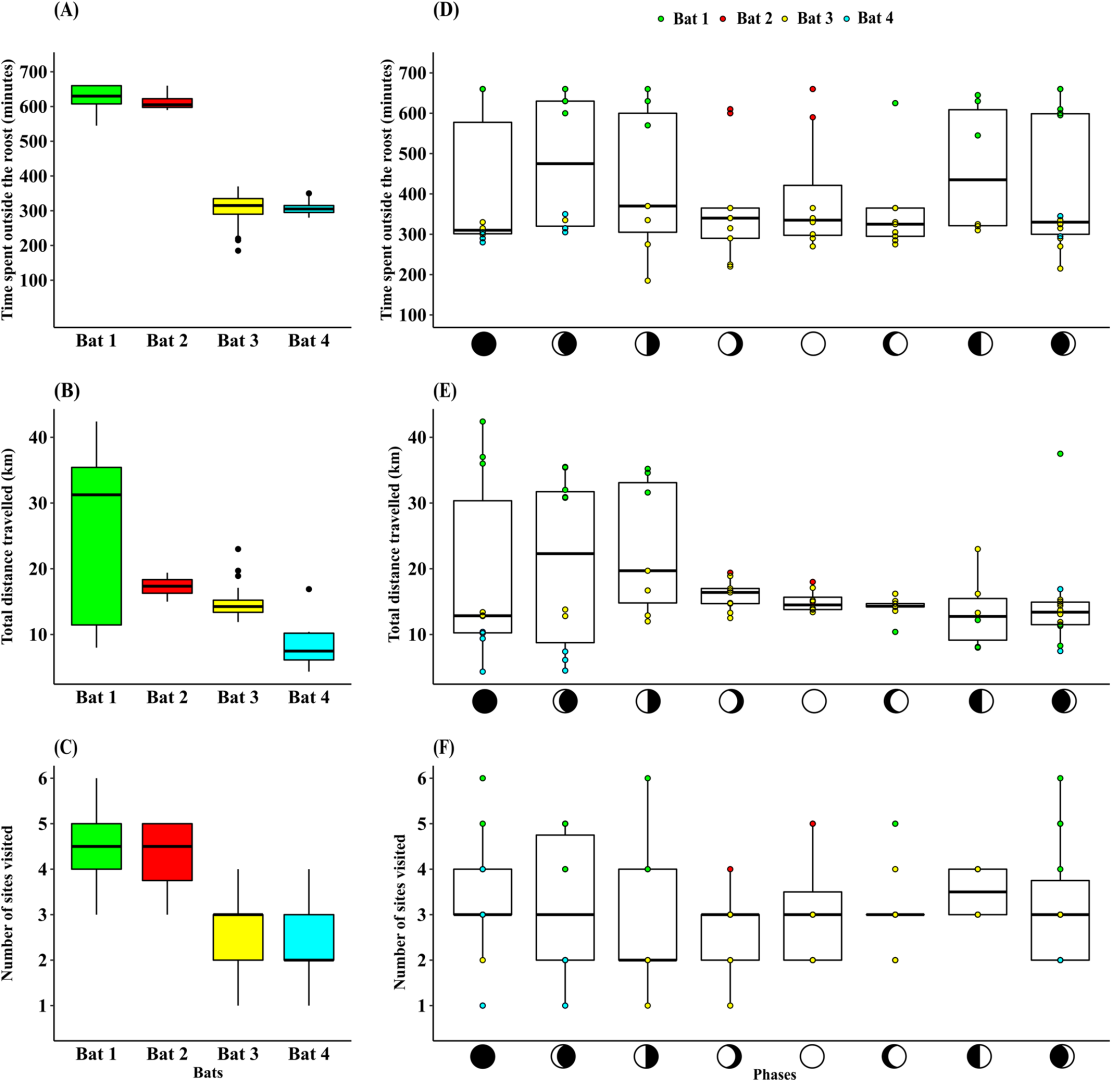
**Bipartite network denoting tree species visited by the five tracked *P.*** ***giganteus*** **individuals**. The breadth of the nodes represents the number of sites in which each plant species was present. *Indicates plant species previously unreported to be visited by *P. giganteus*.

Home ranges varied among the four males, and sub-adult Bat 1 had the largest home range (Fig. 2; Table 1). All individuals had at least one core foraging area which was evident from the 50% kernel density estimates. The number of foraging sites visited per night varied between individuals, from one to six (Table 1; Fig. 2C). These sites covered a range of habitats such as trees in residential areas, trees along highways surrounded by urban areas, fruit plantations, natural vegetation, and isolated trees in agricultural farms (Fig. 1). More than 50 sites were visited by the five tagged individuals, of which 37 were later surveyed. Of the total of 21 recorded tree species (Fig. 3), *Ficus religiosa, Mangifera indica,* and *Manilkara zapota* were frequently visited by four individuals, while *Azadirachta indica, Ceiba pentandra,* and *Tamarindus indica* were visited by three individuals*.* Sub-adult Bat 1 visited the highest number of sites (16) and possibly fed on the fruits and/or nectar of an agave species (at a site localised from the GPS fixes), which has not been reported as a food plant for *P. giganteus* previously.

### Moon phase and individual flight patterns

**Fig. 4.**
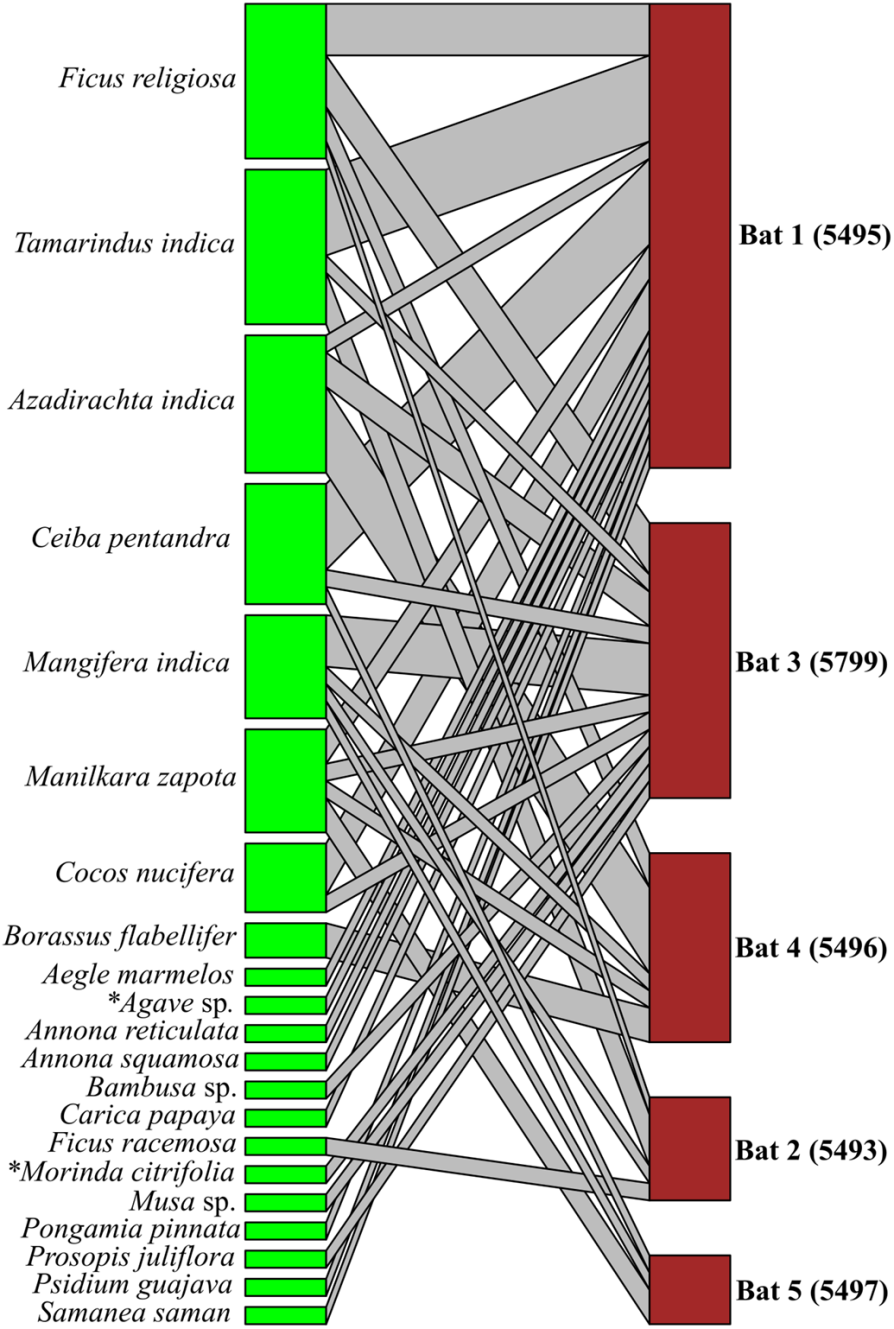
**Nightly inter-individual comparisons of (A) time spent outside the roost, (B) the distance commuted, and (C) the number of sites (*N*=20, 4, 40 and 9 nights for Bats 1, 2, 3 and 4, respectively).** Comparison of (D) time spent outside (E) the number of sites visited, and (F) distance commuted per night by each individual during eight moon phases [*N*=73 nights]. The colours of the data points in panels d, e and f, represent individual bats and symbols in the x-axis denote moon phases: new moon, waxing crescent, first quarter, waxing gibbous, full moon, waning gibbous, third quarter and waning crescent.

Moon phase had no significant effect on the time spent outside the roost per night [Kruskal­–Wallis test, H(7)=4.52, *P*>0.05; Fig. 4D], total nightly distances commuted, [Kruskal–Wallis test, H(7)=7.44, *P*>0.05; Fig. 4E], the number of sites visited per night [Kruskal–Wallis test, H(7)=3.00, *P*>0.05; Fig. 4F]. The variance differed significantly between moon phases, for the distance commuted per night [Levene's test: F(7)=5.08; *P*<0.001; Fig. 2E), but not for the time spent outside the roost [Levene's test: F(7)=0.76; *P*>0.05], or for the number of sites visited per night [Levene's test: *F*(7)=1.25; *P*>0.05].

### Flight directionalities

**Fig. 5.**
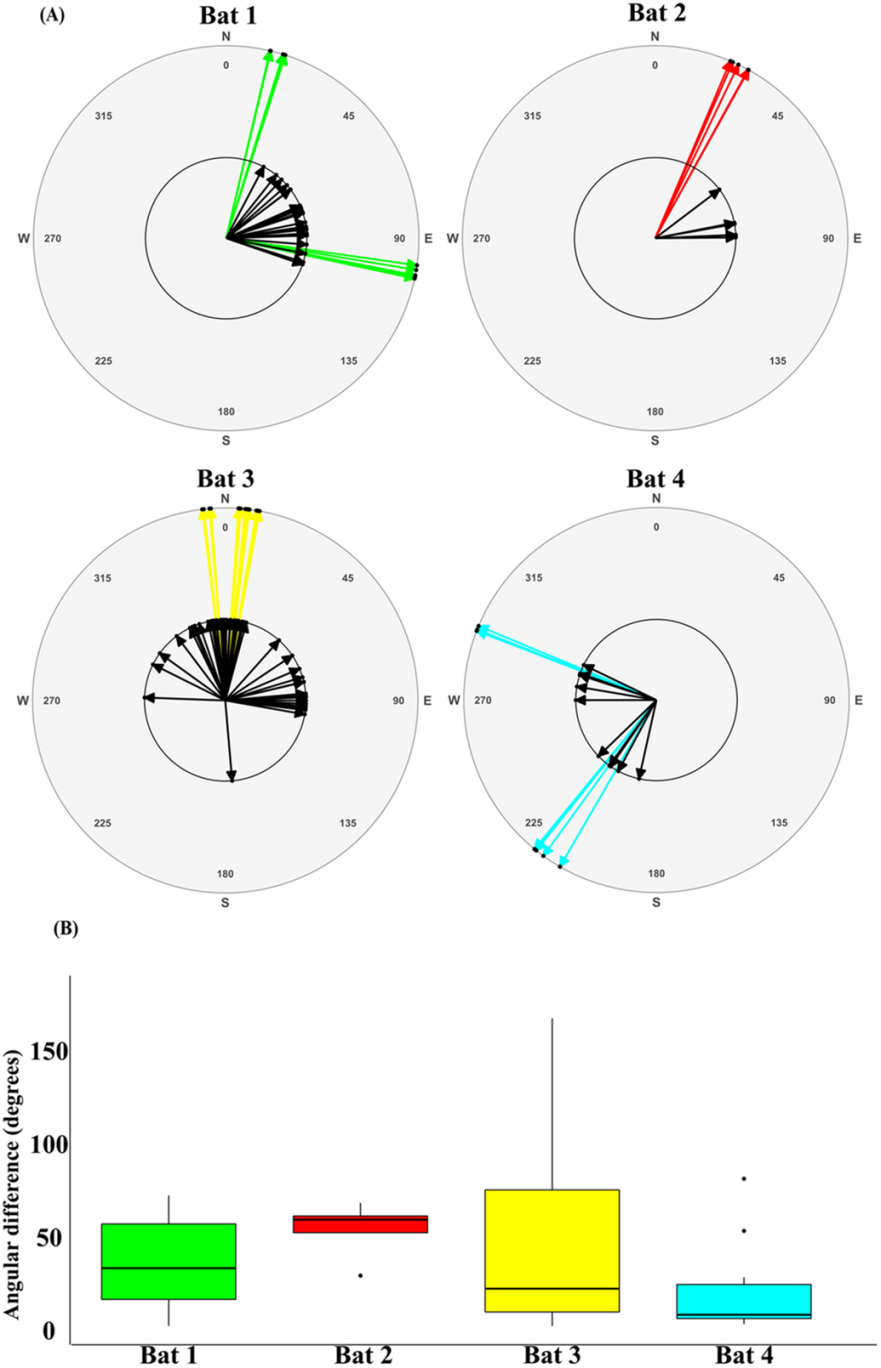
**(A) Angles that were made by the first GPS fix (inner black circles and black arrows) and the first foraging trees (outer circles and coloured arrows) per night relative to the roosting site (centre of circles).** (B) The angular difference between the first GPS fix and first foraging tree relative to the roost for the four study individuals, *n*=88 with 22, 5, 51 and 10 nights for bats 1, 2, 3 and 4 respectively.

The mean±s.d. angular difference between the first GPS fix and the first foraging site of individuals differed between individuals (tracking data, Fig. 5). Adult Bat 2 had the highest angular difference of 52±15 degrees (range: 27–66; *n*=5 nights) followed by adult Bat 3 (40±39; range: 0–165; *n*=51), sub-adult Bat 1 (34±23; range: 0–70; *n*=22) and adult Bat 4 (19±26; range: 1–26; *n*=10; Fig. 5B). In 54 out of 88 nights (61%), the angular difference was below the 45° cut-off for all individuals combined suggesting directional flights from the roost to the first foraging site (Fig. 5A). In addition to this, the sub-adult also visited sites in the eastern side of roost in the first eight observation nights, covering a maximum distance of 12.2 km per night, following which it foraged at sites in the north, up to 20 km from the ninth night (Fig. 1B). Excluding one or two exceptional nights, the three adults maintained consistent foraging directions throughout the study (Fig. 1C-E; Fig. 5A), suggesting individual emergence directions are likely influenced by the foraging locations. Colony-level observations showed that emergence was not equal in all directions relative to the roost as a majority of the colony emerged in the northeast (27%), west (22%) and southwest (19%) collectively (Fig. S2).

### Day roosting activity

Day roosting activity was recorded for 23 days from the four individuals that made at least one successful foraging trip (Fig. S3). There were inter-individual differences in the time spent on any given tree in a day. Bats 3 and 4 consistently (Fig S3C,D) roosted on the same tree during the day (*N*=2.5 days and 4 days, respectively) and returned to it after completing foraging at night (*N*=54 and 10 nights, respectively) whereas Bat 1 (sub-adult) and Bat 2 moved frequently between all four roosting trees (*N*=11, 5 days, respectively; Fig. S3).

## DISCUSSION

By quantifying nightly movement in Indian flying fox males, we found that flight patterns and home range size varied among individuals with the sub-adult covering a larger range than the adults. We also found movement patterns of males were not influenced by moon phase except for a difference in the variance of nightly distance commuted between moonlit and moonless nights. Individual tracking also showed the presence of a foraging site within 45˚ of the emergence direction in 61% of total tracked nights. In addition to the tracked individuals, a noticeable directionality was observed at the colony-level during the 4 months of scan observations. Overall, this suggests that flight behaviour in *P. giganteus* is little influenced by variation in moonlight, and their emergence flights are likely influenced by the directions of foraging resources in their habitat.

Ambient light levels across moon phases can influence flight and visual navigation in nocturnal animals. Smaller frugivorous bats show reduced foraging activity on full moon nights than on new moon nights (Morrison, 1978; Elangovan and Marimuthu, 2001). Sudhakaran and Doss (2012) reported that the total number of visits by *P. giganteus* at foraging sites was lower on full moon than on new moon nights, but suggested that foraging activity was generally not affected by moon phase in *P. giganteus* relative to two smaller sympatric fruit bats, *Cynopterus sphinx* and *Rousettus leschenaultii*. In the same study site, Murugavel et al., (2021) have shown that moon phase had little influence on flight activity of *P*. *giganteus* at the colony-level using roost scans. Here we found that individual movement patterns including time outside the roost, distance commuted and the number of sites visited did not differ with moon phase in tracked individuals.

Being open-roosting and exposed to higher light levels in the day, *P. giganteus* and other flying foxes are thought to be more light-tolerant than other frugivorous bats that show lower flight activity on full-moon than on new-moon nights (Elangovan and Marimuthu, 2001; Morrison, 1978). Also, moonlight avoidance is often hypothesised to be linked to reduced predation in smaller frugivorous bats (Elangovan and Marimuthu, 2001; Morrison, 1978). Owing to their large sizes flying foxes might not suffer much predation from visually guided predators at night, when compared to smaller bats, and could explain not detecting a reduction in flight activity on brightly lit nights in our study. Except for one, all the tracked individuals provided data for less than a complete lunar cycle (Table S1). Hence, we pooled all the data for individuals to compare moon-phase-based patterns. Thus, we could not take into consideration any interindividual variation in this study. However, moon phase seems to have little influence on *P. giganteus* flight activity both at colony (Murugavel et al., 2021; Sudhakaran et al., 2012) and individual levels.

Flying foxes and other gregarious tree-roosting pteropodids have larger foraging ranges than smaller pteropodid bats (Acharya et al., 2015; Aziz et al., 2021; Bonaccorso and Gush, 1987; Marimuthu et al., 1998; Winkelmann et al., 2000), allowing them to act as long-distance pollinators and seed dispersers (Aziz et al., 2017; Aziz et al., 2021). Resource availability influences annual movement in several pteropodid species (Eby, 1991; Hurme et al., 2022; Richter and Cumming, 2005; Roberts et al., 2012). Even though there were inter-individual differences in the flight directionalities (Fig. 5), the emergence directions of all tracked individuals seemed predominantly to be aligned with the direction of the first foraging site, suggesting an effect of foraging resources on their emergence flights. Thus, the colony-level directional preferences could potentially be used as an indicator of resource availability and distribution.

During the study, we lost three of the eight tagged bats within 10 days, including Bat 4, which was found at a different roost. This happened during the time of year when mango trees, one of the most visited plant species during the study, stopped fruiting in the study landscape. More specifically, Bat 4 was found at the other roosting site ∼108 km away during the peak of guava and jackfruit season. Within the study, period Bat 1 shifted from eastern foraging sites to northern foraging sites after eight nights, probably because of a change in the availability of resources. Hence, it is possible that the availability of food resources in the landscape can trigger shifting of roost (at least in males) and/or foraging sites in *P. giganteus,* as is known in other flying foxes (Eby, 1991; Palmer and Woinarski, 1999).

Differences in home range and nightly distance travelled among adults and sub-adults have been reported in *P. niger* between the summer and winter months (Oleksy et al., 2019). Similar to *P. giganteus* in this study, sub-adults of *P. dasymallus* had larger home ranges than adults (Nakamoto et al., 2012) and juvenile *P. tonganus* performed long-distance exploratory flights compared to distances travelled by adults (Banack and Grant, 2002). Nakamoto et al. (2012) suggests that low experience in the landscape could lead to such exploratory flights by sub-adults. Returning to the same sites for multiple nights is a more energy-efficient foraging mode than random foraging (de Jong et al., 2013) but requires more experience in the landscape, which adults likely possess. By tracking Egyptian fruit bat (*Rousettus aegyptiacus*) pups during their initial flights, Harten et al. (2020) found that home ranges of young individuals gradually expanded until they reached adulthood as they learned locations of new foraging sites. In this study, Bat 2 and Bat 3 visited the same foraging sites throughout the study period, suggesting established foraging routes and locations. Such site fidelity was specifically prominent in Bat 3. When it diverged from its usual northward route due to strong winds, it travelled with the wind westward along the mountain range (elevation ∼300 m above sea level), and took a northward turn at a gap in the mountain range, to reach its usual foraging trees (Fig. 1D; Fig. S4). Recent movement studies on Egyptian fruit bats support visual map-based navigation, which could possibly explain inter-individual differences (Harten et al., 2020; Toledo et al., 2020). While this might also explain the inter-individual differences observed in this study, previous flight experience of individuals is unknown which limits the interpretation of these differences.

Variation in colony sizes usually serve as indicators of emigration or immigration in gregarious fruit bats (Parry-Jones and Augee, 1992). In *P. giganteus,* colony sizes vary across the year, reaching a peak during the breeding season (Mathur et al., 2012; Mishra et al., 2020). Short-range shifts from a central roost to secondary roosts for 2 to 3 days have been reported in male *P. giganteus* in Myanmar (McEvoy et al., 2021). There were at least four known roost sites within a 20 km radius around my study colony but we did not find any tracked individuals in these alternate roosts after they went missing from the study roost. However, we found that an adult male moved to a second roosting site ∼108 km away, indicating long-distance movement between colonies in *P. giganteus* males. Based on the information from *P. giganteus* males from my site and from Myanmar (McEvoy et al., 2021), we suggest that a possible reason for the variation in colony sizes could be the movement of males between roosts for the purpose of mating. However, this hypothesis requires confirmation from more roosts and individuals.

Neuweiler, (1969) reported a vertical rank order in occupying roosting locations on the tree among *P. giganteus* males which was correlated with social dominance*.* From our limited observations on four males at the day roost, it appeared that adult Bats 3 and 4 had more consistent roosting positions than sub-adult Bat 1 and adult Bat 2 (Fig. S3). Bat 2 showed some similarities with the other two adult males (e.g. restricting its foraging to sites closer to the roost) but in other aspects, its behaviour was similar to that of the sub-adult Bat 1, such as not having a fixed roosting position and visiting more foraging sites per night. This individual was classified as an adult based on prominent testicles but it had the lowest body weight among all adult bats in the study (Table S1). It is possible that this individual was a young adult which was still in the process of exploring and learning the landscape. Australian *Pteropus* spp. are known to be nomadic throughout the year, and males constantly shift their roosts over time (Roberts et al., 2012; Welbergen et al., 2020). An ‘old’ male among our study individuals was found roosting on different foraging trees solitarily on two different days and never returned to the colony suggesting that old males might move nomadically between roost sites.

Flying fox colonies occur in close proximity to human habitation across the distribution range, and urbanisation has influenced their roosting and foraging patterns (Choden et al., 2019; Meade et al., 2019; Timmiss et al., 2021). Electric lines pose a direct threat to the conservation of flying foxes as they have been identified as one of the major causes of mortality in Australia, India, and Sri Lanka (Tidemann et al., 2021; Chouhan and Shrivastava, 2019; Tella et al., 2020). Two out of the four study individuals from which we retrieved the transmitters were found to be electrocuted (Table 1), suggesting that this is a serious problem. Further investigations focussing on flight patterns, heights, and commuting routes can help in evaluating fatalities due to electrocution and potentially aid the conservation of the species. In human-dominated landscapes, flying foxes prefer foraging in residential areas over plantations (Choden et al., 2019), and it is important to identify and conserve such feeding sites that are crucial for the sustenance of the species in these landscapes. My study has located more than 50 sites that were visited by tagged individuals in the semi-urban study landscape*.* Even though the study colony was situated in a semi-urban landscape, a significant part of the home ranges of tagged individuals was in the forest and natural vegetation.

Overall, our results suggest that the movement patterns of Indian flying foxes are little affected by variation in ambient light levels, while resource location and availability determine emergence flight directions. However, it is still not known how individuals use areas with varying levels of artificial lighting and how anthropogenic changes influence their movement. For a species occurring throughout the Indian mainland, including in the megacities (Mishra et al., 2020), an understanding of space use and foraging trends in these landscapes is crucial, especially in the context of light pollution and zoonotic pandemics. In general, flying foxes are key pollinators of economically important fruits such as *Durio zibethinus* in Malaysia and are key long-distance seed dispersers across the paleotropics (Aziz et al., 2017; 2021), and *P. giganteus* visits many commercially important trees across the Indian subcontinent. However, their role as seed dispersers and pollinators in these landscapes is still unclear and requires further investigation.

## MATERIALS AND METHODS

### Study site

We captured bats from a colony of *Pteropus giganteus* at the Centre for Biodiversity and Forestry Studies, Madurai Kamaraj University, Madurai (9°56′24.01″N and 78°1′0.84″E) in southern India from February to September 2019. The colony roosted close to a mountain range (Fig. S5) on four neighbouring trees of *Albizia lebbeck* and *Ficus benghalensis*, two of which were main roosts occupied by the bats when seasonal population size was low. The colony size varied from 2000 to 9000 individuals during the study and the habitat within a radius of 5 km of the roost consisted of a mix of human settlements, natural vegetation, farmland, and open rocky terrain.

### Animal capture

Eight *P. giganteus* males were captured between February 2019 (beginning of breeding season) and September 2019 (post-breeding season), using customised mist nets (15 cm mesh size) installed near the roost using 10-12 m high bamboo poles. Bats were trapped during emergence or while returning to the roost. The only two females that were trapped had pups and were released immediately. Thus, only males were tracked during this study. Approval for field studies was obtained from the National Biodiversity Authority of India (NBA/Tech.Gen/22/145/15/ 16-17/2561). All animal handling and field protocols were approved by the Institutional animal ethics committee (IAEC) of the Indian Institute of Science Education and Research Thiruvananthapuram (IISER TVM).

### GPS Tagging

After removing bats from the mist net, they were moved into an observation cage (3×3×3 m^3^) where solar-powered GPS transmitters (15 g, e-obs GmbH, Gruenwald, Germany) were carefully attached to them using a customised 3D-printed collar backpack with a weak link around the neck (Fig. S6A, B). The entire setup weighed between 25 g and 35 g constituting less than 5% of the body weight of the individuals, which is within the accepted weight limit for tracking studies in bats (Aldridge and Brigham, 1988; Amelon et al., 2009; O'Mara et al., 2014). The animals were fed with fruit juice to keep them hydrated. Animals captured during emergence were observed overnight (Fig. S6C), while those captured on their return flight were observed for 30 to 60 min and released before 07:00 h (local time). Animals were provided with bananas and grapes hung inside the cage. Morphometric measurements, body weight, and the reproductive status of the animals were recorded before collar attachment. Following Racey (2009) males with body mass over 700 g and conspicuous testes were classified as adults (*n*=7), and others as sub-adults (*n*=1). An adult that had missing teeth, wrinkled skin, and punctured wing membranes was classified as ‘old’ male.

### Tag settings

Tags were programmed to provide GPS fixes every 30 min when the animal was resting (low resolution) and every 5 min during flight (high resolution). The switch-over from low to high resolution was programmed to start once the animal crossed a variance threshold speed of 50 cm/s. Tri-axial acceleration data were collected at 30 s intervals with a byte count of 316 (16.67 Hz/axis). Tags were programmed to be on for the entire day (24 h) during the initial 2 to 11 days to record both daytime roost and nocturnal foraging activities (Table S1). For Bat 1, the tag was kept on for 24 h for the first 11 days. However, due to high battery power consumption and insufficient recharge rates (solar power), we switched to keeping the tag on for 24 h over for only 2-4 days for the remaining bats (see Table S1 for details). After this period, tags were re-programmed to provide positions only between 18:00 h and 07:00 h to record nocturnal activity. The loggers also had an ultra-high frequency (UHF) pinger, which was programmed to be active between 10:00 and 11:00 h for localising the animal in the roost using the UHF radio link. These settings were modified slightly for each individual depending on the battery power and status. After deployment of the GPS transmitter, roosts were scanned daily to locate the tagged individual using a wide range radio receiver (AR8200 Japan) attached to a Yagi antenna and by visual observations whenever possible (Fig. S6D). Data were downloaded using the handheld base station (e-obs GmbH, Gruenwald, Germany), and transferred to a personal computer. The logger.bin file was decoded using the decoder (Version.7.5) downloaded from the e-obs website (https://e-obs.de/).

### Tracking data and field surveys

The GPS positions were plotted in Google Earth Pro (version 7.3.3.7786) and used to calculate the nightly distance travelled by each bat. A foraging site was defined as a location where a tagged individual spent more than 15 min (corresponding to at least three high-resolution fixes or two low-resolution fixes). Five of the eight tagged bats provided data for more than one night (Table 1). Based on tracks from the GPS positions, a successful foraging trip on a given night was defined as a complete round trip, from the starting position when the first fix was obtained (emergence initiation), multiple positions at sites (visitation/foraging), to the final position back in the roost (flight termination). The difference between the emergence initiation and the flight termination times was the total time spent outside the roost on a given night. For each night, the total distance of the tracks connecting all the fixes was calculated as the total nightly distance travelled. Nightly movement information was compared among four individuals that had completed more than one successful foraging trip. However, since the GPS fixes were obtained at 5-min intervals during flight, the actual distance flown by an animal may be underestimated. Based on the definition of a foraging site (see above), the number of foraging sites visited per night was estimated. Depending upon battery power, data obtained were incomplete on some nights for all four bats (Table 1). Such nights of partial data collection were excluded for calculating the nightly distance, time spent outside, and number of sites visited.

In the weeks after tracking of each individual was completed (either after the animal went missing or the transmitter was retrieved), these sites were localised on Google Earth Pro using GPS fixes and surveyed wherever possible. The tree species were determined and listed as plants visited based on GPS fixes. On some occasions feedings signs were observed at these sites to confirm foraging activity.

### Moon phase and nightly movement

The total nightly distance commuted, number of sites visited and time spent outside the day roost (minutes) per night were examined in relation to moon phase using the pooled data from all four individuals (*N*=73 entire nights). Data were pooled because individuals varied strongly in the number of nights they could be tracked before the tag was lost. Thus, no statistical tests could be performed on this dataset to account for inter-individual effects. Lunar cycles were classified into phases based on percent illumination (obtained from www.timeanddate.com) as new moon (0-5%), waxing/waning crescent (5.1-34.9%), first/third quarter (35-65%), waxing/waning gibbous (65.1-94.9%) and full moon (95-100%).

### Individual flight directionalities

To examine the flight direction that individuals took from the roost to the first foraging site, we used flight tracks from the first fix out of roost (F_e_) to the first fix at the first foraging site (F_f_) in a given night (*N*=88 nights from four individuals). Using measurement tool in Google Earth, the angles made by F_e_ and F_f_ relative to the roosting site were obtained, and the angular difference between them was estimated for each night. A cut-off of 45° angular difference was set for estimating how aligned the emergence flights were with respect to the first foraging site on a given night. The cut-off was set to divide the area around the roost into eight equal angular sectors.

### Emergence directionalities at the colony-level

In addition to GPS tracking of single males, colony-level observations were carried out in the same study colony between April and July 2019 from 18:00 h to 07:00 h (16 nights in total, of which three were excluded due to bad weather). Observations were made on new moon, first quarter, full moon, and third quarter phases of four lunar cycles using the methods described by Murugavel et al., (2021). In brief, roost observations were made by three observers at a distance of ∼200 meters from the roosting tree in the northwest, south, and east directions. The number of bats emerging from the roost in eight directions (north, northeast, east, southeast, south, southwest, west, and northwest) were estimated every 5 min until the last bat emerged (emergence termination).

### Daytime roost activity

Day roosting information was also obtained from five individuals and roosting habits were compared among the four individuals that made successful foraging trips (Table S1). Based on daytime GPS data (07:00-18:00 h), the proportion of time spent on each of the four adjacent roosting trees on a given day and during the total observation period was compared between individuals.

### Analyses

Data were analysed using R version 4.0.3 (R Development Core Team, 2020). Home ranges for the four tracked individuals were determined using the *sp* (Bivand et al., 2013; Pebesma and Bivand, 2005) and *adehabitatHR* (Calenge, 2006) packages. Minimum convex polygons (MCP) were generated using the function ‘mcp’ to estimate the overall home range, and kernel density estimates (95% and 50% KDE) were calculated using the function ‘kernelUD’. A bipartite network was constructed using the *bipartite* package (Dormann et al., 2008) to visualise the tree species visited by each individual with the weights of the interactions being the number of sites in which a given tree species was present. The nightly movement dataset was not normally distributed and separate Kruskal–Wallis rank sum tests were performed to test whether the total distance commuted, sites visited and time spent outside the day roost varied between moon phases. To test whether the variances differed, separate Levene's tests were performed on the three nightly parameters against the moon phases.

## Supplementary Material

10.1242/biolopen.059513_sup1 Supplementary information
