# Supplementary material for: Home ranges, directionality and the influence of moon phases on the movement ecology of Indian flying fox males in southern India
Source: Biol Open. 2023 Jan 30;12(2):bio059513. doi: 10.1242/bio.059513 (PMC9922730; doi:10.1242/bio.059513)
Supplement: Supplementary information [file biolopen-12-059513-s1.pdf]

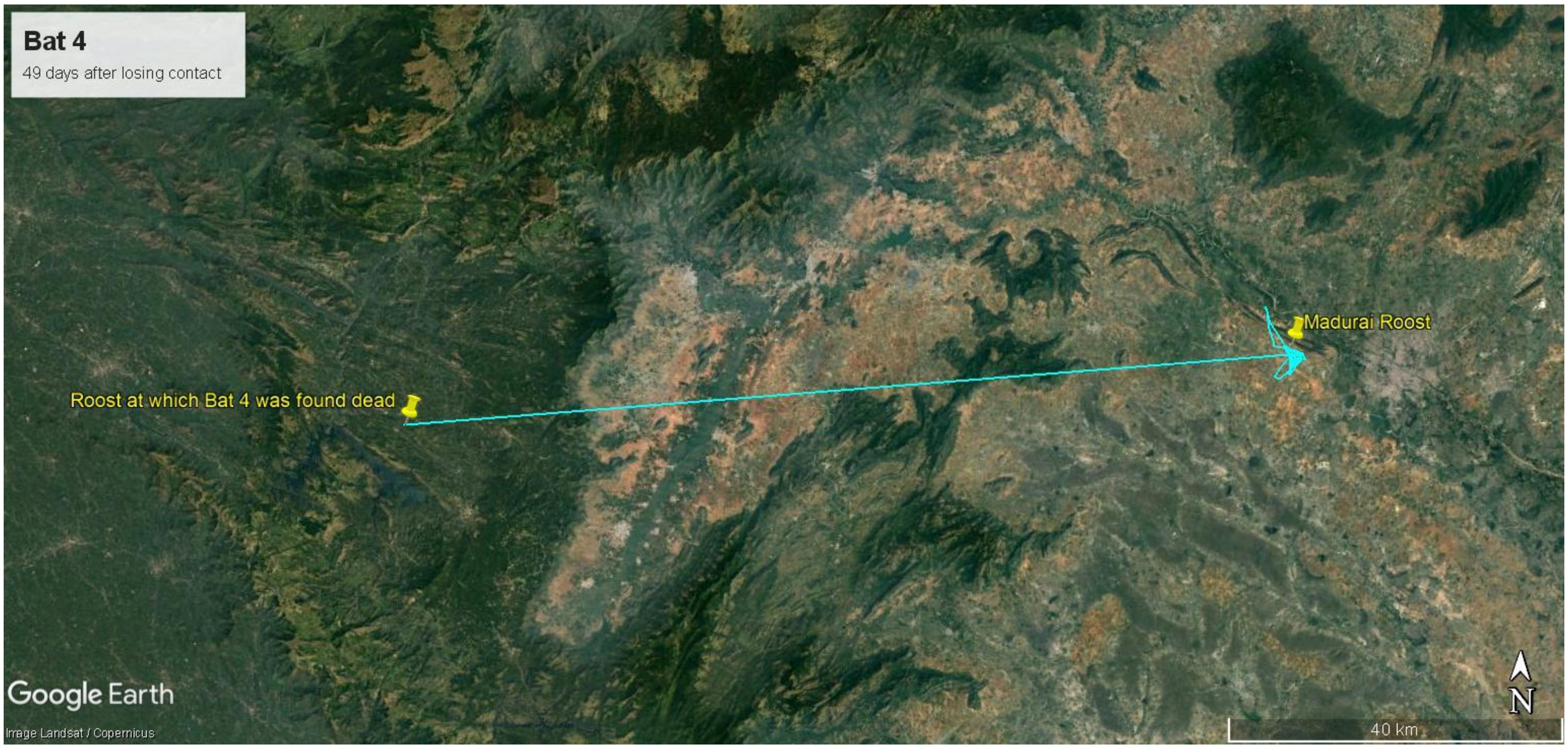

Fig. S1. Long-distance movement by Bat 4. The blue line connects the location of the roost in which it was tagged and the roost where it was found dead. It represents the shortest distance between the two locations (108 km) and not the animal’s flight path.

Biology Open • Supplementary information

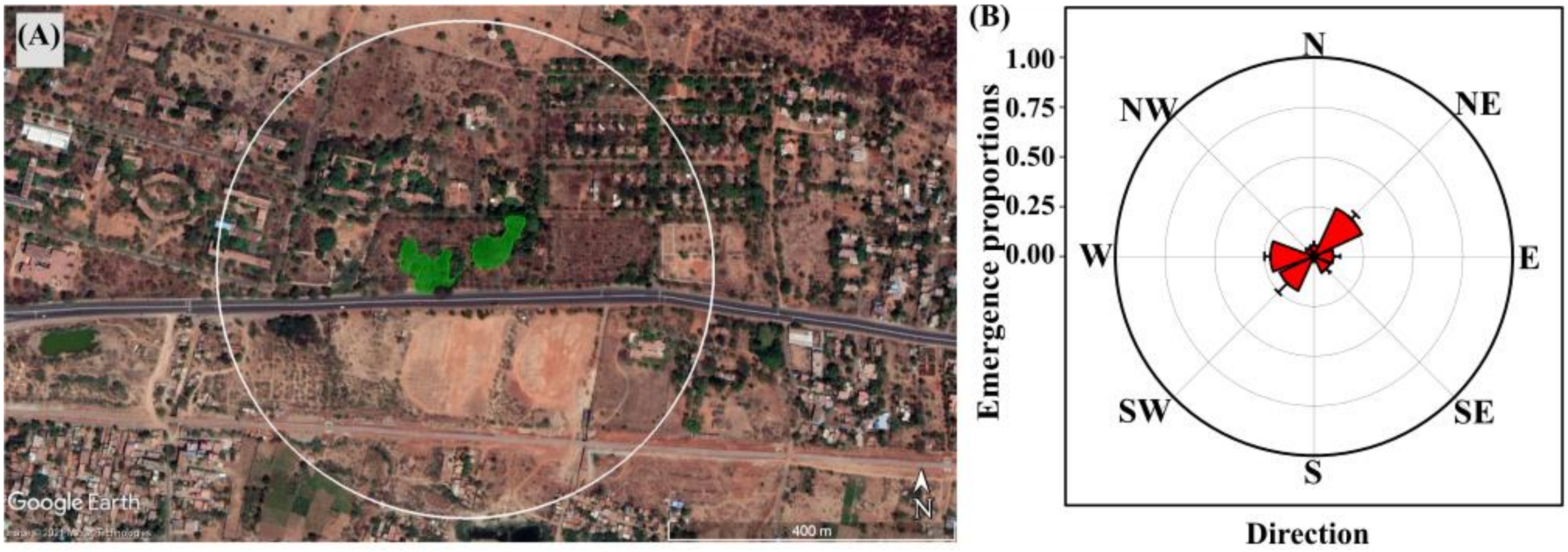

Fig. S2. Directions of emergence of individuals from the study colony. (a) Map showing the roosting trees (green) and the area around the roost that was observed for noting down emergence directionality. (b) Overall emergence directionality proportions (mean) of the colony [n=13 nights]. Error bars represent standard errors.

Biology Open • Supplementary information

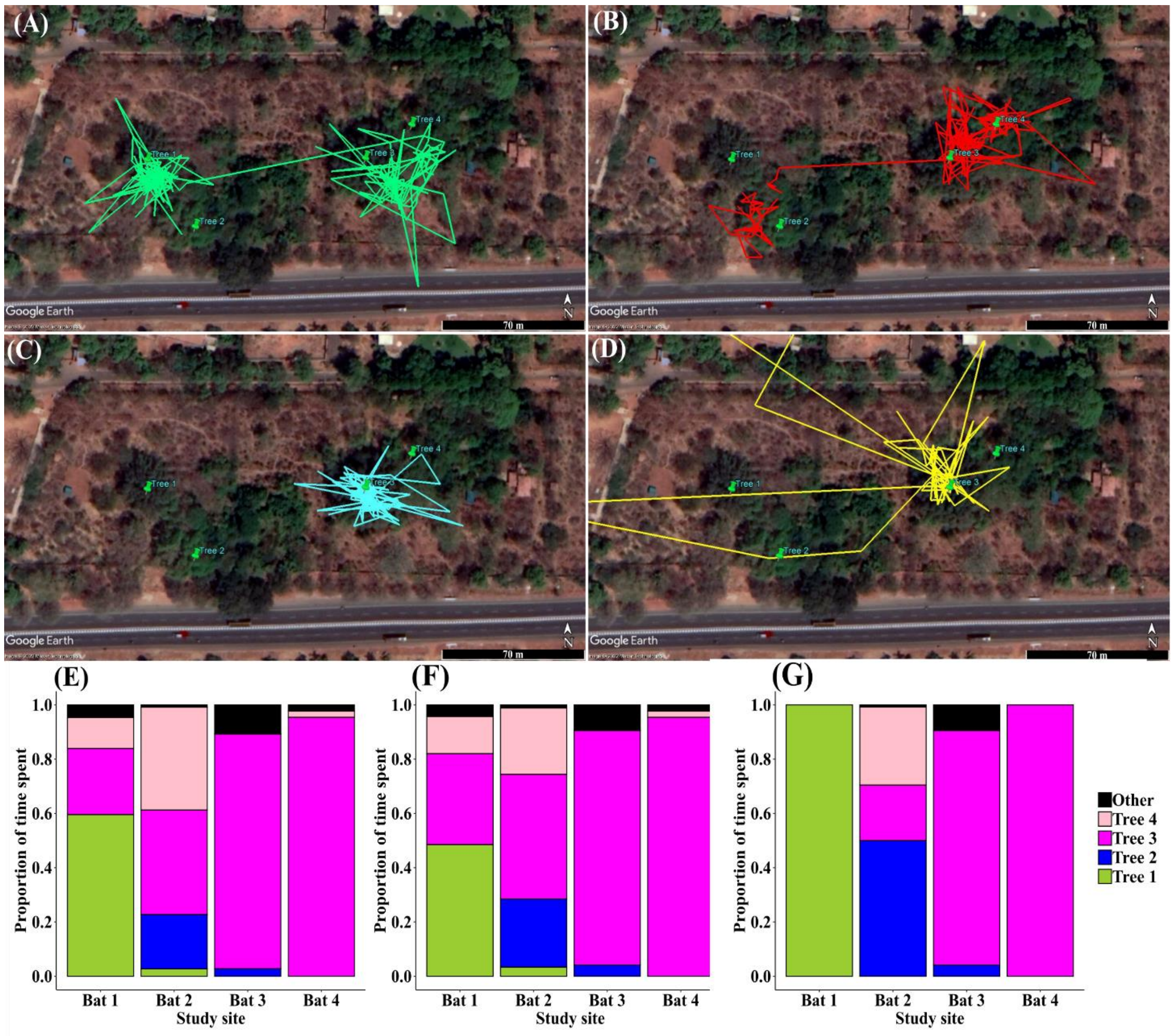

Fig. S3. Day roosting patterns of four tracked individuals (A) Bat 1 (B) Bat 2 (C) Bat 3 (D) Bat 4. (E) Proportions of total time spent by Bats 1, 2, 3, and 4 on each roosting tree during the day [N=11, 5, 4, and 3 days, respectively], (F) from days with data from 07:00 – 18:00 h [N=8, 4, 2 and 4 days, respectively and (G) data collected during the first two days after transmitter attachment. ‘others’ refers to time spent in short daytime flights.

Biology Open • Supplementary information

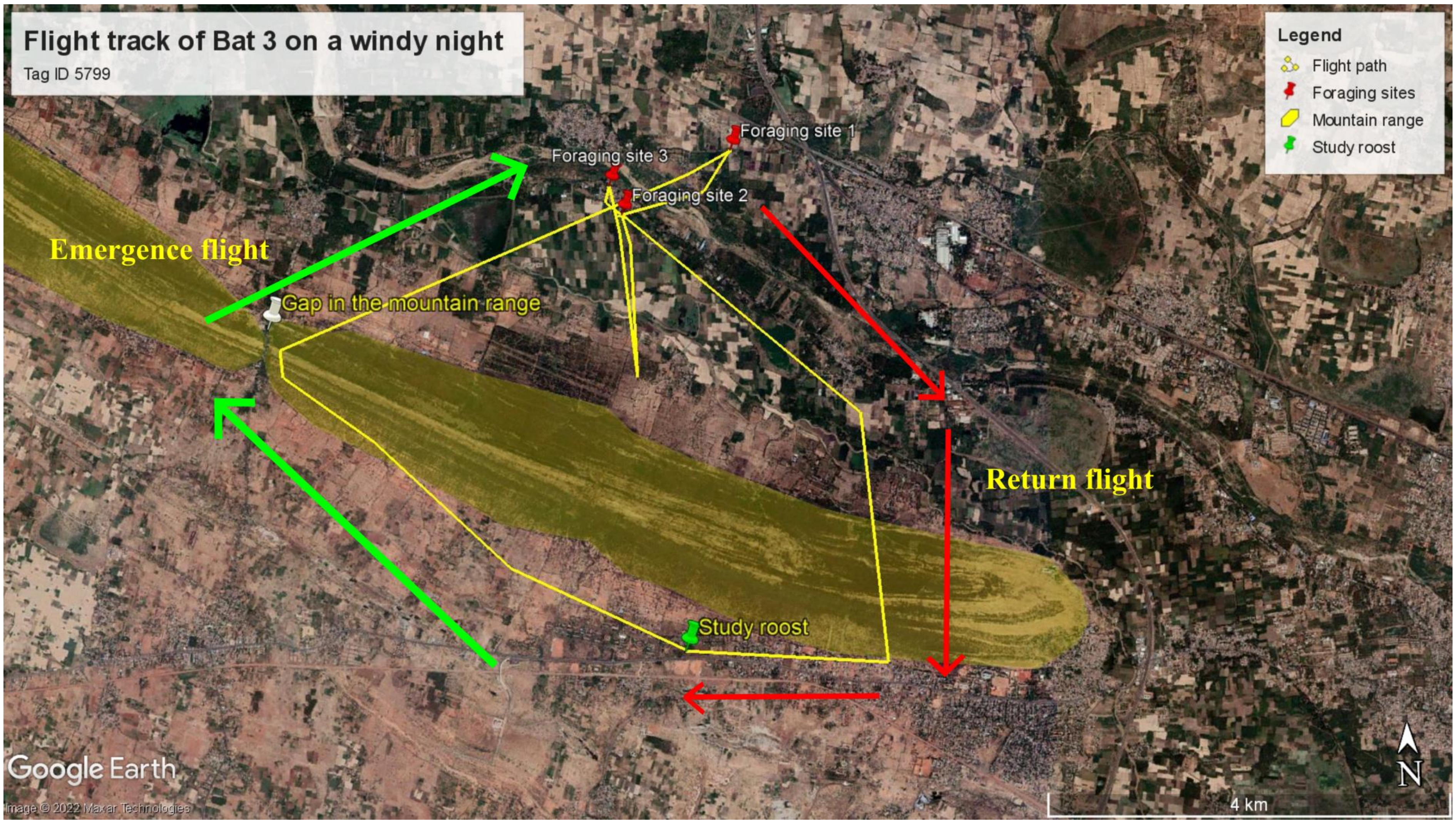

Fig. S4. Foraging track of adult Bat 3 during a windy night. The area shaded in yellow depicts the mountain range (elevation ~ 300 m above sea level) extending to the north-west of the roost. Unlike every other night (Fig. 1d) when the bat took a direct path over the mountain range, on the windy night, it flew Westwards (indicated by green arrows), then took a right turn to pass the mountain gap and visited the same foraging trees that it had visited on previous nights, situated in the north of the roost and returned via its regular route (red arrows).

Biology Open • Supplementary information

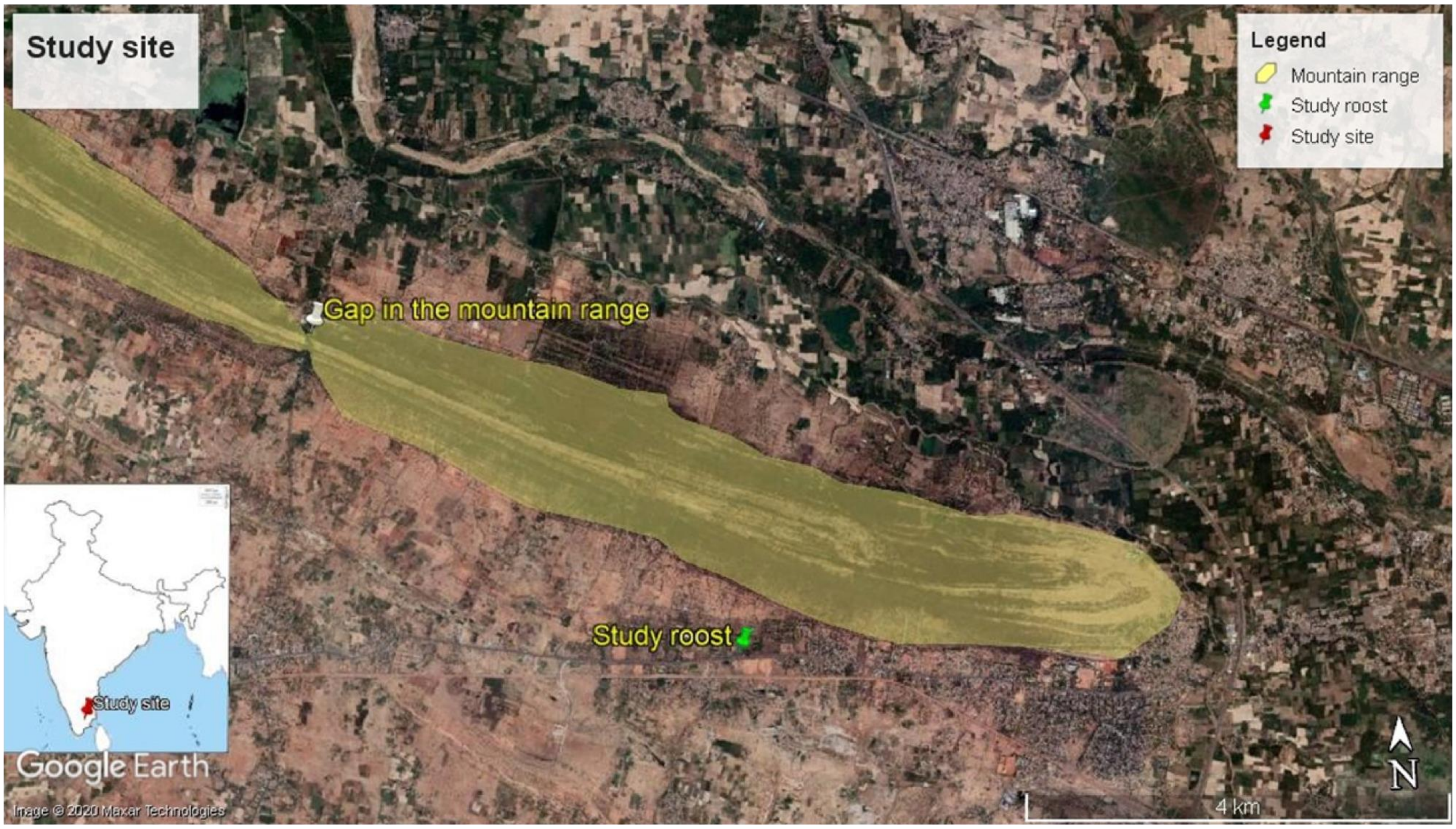

Fig. S5. Map representing the location of the study site (red pointer) and study roost (green pointer). The polygon shaded in yellow denotes the mountain range (elevation ~300 m above sea level) situated north of the colony (~100 m above sea level) and extending ~4 km to the east and ~30 km to the west of the roost with a gap (white pointer) ~4 km from the roost in the north-west direction. India outline from d-maps.org.

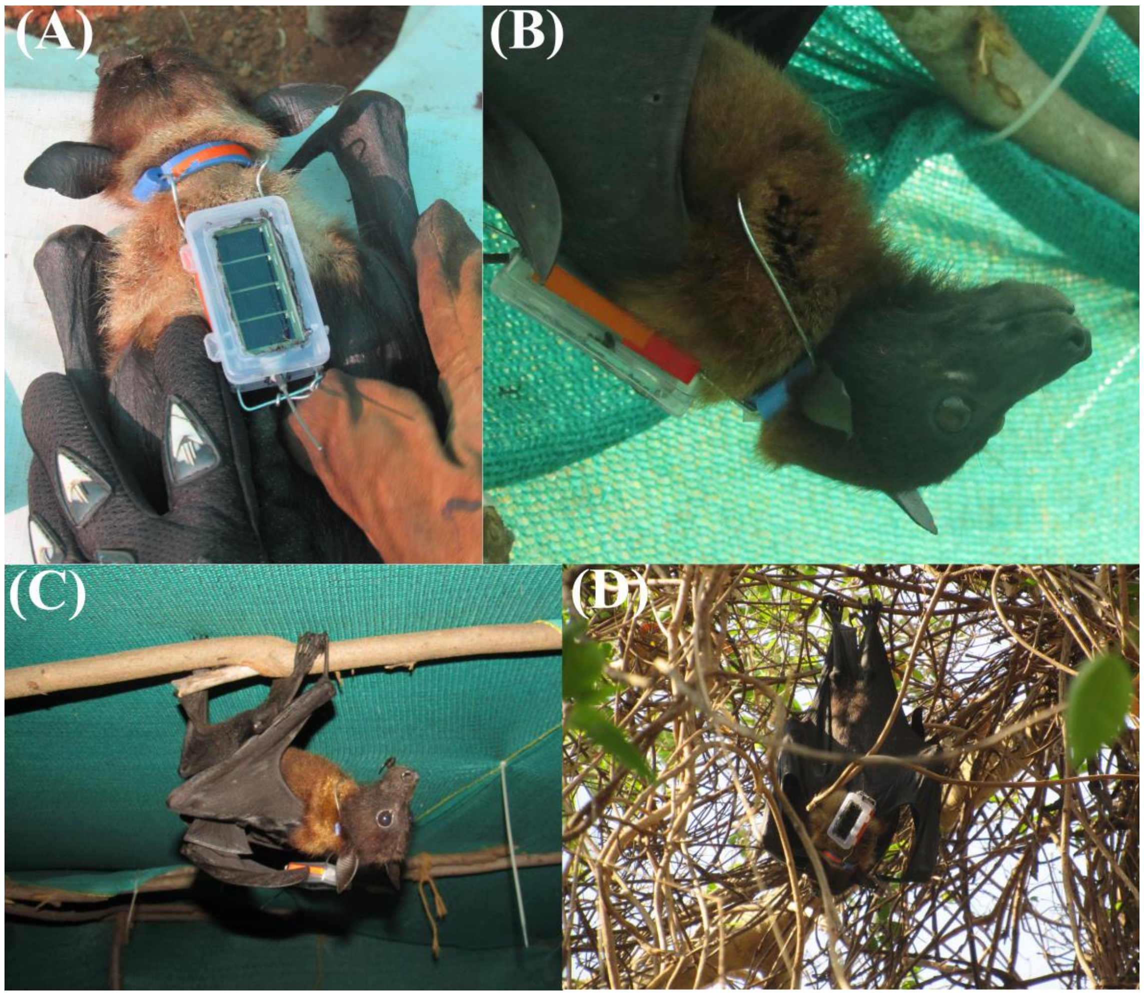

Fig. S6. Transmitters attached to individuals of *P. giganteus* using a customised setup. (A) Top view, (B) side view, (C) a tagged bat inside the cage during overnight observations (D) A tagged bat at the day roost.

Table S1. Details of all the tagged animals during the study.

Tag ID	Sex	Status	Weight (g)	Forearm (mm)	Tagging Date	Date of last obtained fix	Number of nights and days for which data were retrieved* (Complete + partial)		Transmitter status
							Nights	Days	
Bat 1 (5495)	Male	Sub Adult	585	160	23-02-2019	20-03-2019	22 (20 + 2)	11 days (8+3)	Lost
Bat 2 (5493)	Male	Adult	700	170	15-04-2019	21-04-2019	5 (4+1)	5 days (4+1)	Retrieved[a]
Bat 5 (5497)	Male	Adult (old)	885	180	28-04-2019	30-04-2019	3* (2+1)	3 days (2+1)	Retrieved[b]
Bat 6 (5499)	Male	Adult	890	168	05-05-2019	06-05-2019	1* (1+0)	1 day (1+0)	Retrieved[c]
Bat 7 (5499_1)	Male	Adult	685	185	15-05-2019	15-05-2019	0	1 day (1+0)	Lost
Bat 3 (5799)	Male	Adult	875	170	09-06-2019	19-08-2019	54 (40 + 14)	3 days (2+1)	Lost
Bat 4 (5496)	Male	Adult	780	176	28-06-2019	14-08-2019	10 (9+1)	4 days (4+4)	Retrieved[d]
Bat 8 (5798)	Male	Adult	775	168	23-08-2019	24-08-2019	0*	1 day (1+0)	Lost

*****Captured during return flights and were not observed overnight.
[a] Animal found dead on an electric line near a foraging site; [b] Animal was recaptured from a foraging site during the day for retrieval;
[c] Animal predated by a dog at the roost
[d] Animal found dead at a second roosting site ~108 km away from the study roost. Cause of death: Found on an electric line

Biology Open • Supplementary information
